# A comparative study of mental health and wellbeing in adopted children, children in foster and kinship care, and the general population

**DOI:** 10.1186/s12887-026-07104-x

**Published:** 2026-08-21

**Authors:** Edward Janes, Amy L. Paine, Rebecca Anthony, Bethan Carter, Katherine H. Shelton

**Affiliations:** 1 School of Psychology, Cardiff University Centre for Human Developmental Science, Cardiff University, Tower Building, 70 Park Place, Cardiff, CF10 3AT United Kingdom; 2 Centre for Development, Evaluation, Complexity and Implementation in Public Health Improvement (DECIPHer), School of Social Sciences, SPARK, Cardiff University, Cardiff, CF24 4HQ United Kingdom

**Keywords:** Adoption, Foster care, Kinship care, Mental health, Wellbeing, Adolescents, Care-experienced children

## Abstract

**Background:**

Children with care experience are at elevated risk of poor mental health and wellbeing, yet large-scale comparative studies of placement type, particularly including adoption following a period in care, remain limited. This study examined mental health and wellbeing outcomes across different care arrangements using population-level data from children attending secondary school (age 11 to 16) in Wales, UK.

**Methods:**

Data were drawn from the School Health Research Network 2023 Student Health and Wellbeing Survey, completed by 129,761 children across 202 mainstream schools in Wales, UK. Analyses included 103,316 children in the general population and 2384 care-experienced children, comprising those in kinship care (*n* = 1,460), non-relative foster care (*n* = 589), and adoptive families (*n* = 335). Groups were compared on youth self-reported measures including the Strengths and Difficulties Questionnaire (SDQ), Short Mood and Feelings Questionnaire (SMFQ), and the Short Warwick-Edinburgh Mental Wellbeing Scale (SWEMWBS).

**Results:**

Children in the general population had better mental health and wellbeing outcomes than care-experienced peers. Adopted children reported the poorest mental health outcomes across all domains, exhibiting significantly higher total SDQ difficulties, internalising and externalising difficulties, depressive symptoms, and the lowest wellbeing scores. Children in foster care had better mental health and wellbeing relative to the other care experienced groups, followed by children in kinship care.

**Conclusions:**

Findings highlight substantial disparities in mental health and wellbeing among care-experienced youth, with adopted children showing the most elevated difficulties. These results underscore the need for policies to include targeted mental health support for care-experienced children across all settings, including post-adoption contexts.

**Supplementary Information:**

The online version contains supplementary material available at 10.1186/s12887-026-07104-x.

## Background

Children in care face significantly greater risks of poor mental health outcomes compared to those raised in private households [1]. This disparity is often attributed to the higher likelihood that children entering care have experienced early-life adversity, including maltreatment or neglect [2, 3]. These adversities have been theorised and found to have the capacity to exert lasting effects on emotional, behavioural, and cognitive development for those children who leave care via adoptive placements [4]. Understanding the mental health and wellbeing of care-experienced children, therefore, remains a critical challenge.

Ford et al. [5] conducted a landmark UK study comparing children in care (aged 5–17) with those not in care, showing significantly higher rates of mental health difficulties and neurodevelopmental conditions among the care-experienced group. Subsequent research has reinforced these disparities, with children (under 18) in care twelve times more likely to experience mental health problems than those with no social services involvement [6]. In the 2017 School Health Research Network (SHRN) survey (Wales, UK), secondary school-aged children in care reported lower wellbeing compared to the general population [7]. Furthermore, earlier SHRN findings with the same age group linked being in foster care to increased substance misuse and lower life satisfaction [8]. A meta-analysis [9] also found suicide attempts to be over three times more prevalent among children in care. Long-term effects are also evident in adulthood, with care history associated with poorer mental health outcomes [10]. Although this research indicates that children and adolescents in care are consistently found to be at heightened risk for mental health difficulties, population studies of prevalence or impact with large sample sizes remain scarce [11, 12].

### The role of placement type

For children who enter the care system, there are an additional set of contextual factors that need to be considered pertaining to their time in care. One central factor is the type of placement, and a meta-analysis of studies from around the world [13] found that children and young people (under 21) in foster care exhibited elevated levels of both internalising and externalising problems compared to those living in private homes. However, when comparing placement type, children in kinship care had more favourable mental health outcomes compared to those in non-relative foster care. Additional studies have found that placement type may play a significant role in mental health conditions for children and young people under the age of 21 [14]. This includes children in kinship care (who had a mean age of 4) having better wellbeing and a lower rate of mental health conditions and behavioural difficulties than those in non-relative foster care [15]. A systematic review of resilience in predominantly high-income countries also found that children (under 19) in residential care had lower wellbeing than other children, though those in this setting who were better able to cope with trauma were more likely to have better developmental outcomes [16]. Overall, these findings point to the potential importance of placement type in mitigating psychological risk for children who cannot live with their birth families. There is also evidence that it is actually one of several factors relating to the lives of children (under 18) before, during or after their time in care that cumulatively affect their later lives [17].

### Adoption

Despite being care-experienced, the mental health and wellbeing of children adopted from care have rarely been studied in the context of large scale, population-based research. This has limited opportunities for researchers to compare adopted children and children with those who are living in the general population or currently in care. One exception is Carter et al. [18] who profiled different care-experienced children aged 10–17 in the UK. Carter found that mental health and wellbeing outcomes for all participants tended to worsen with age, but that children in non-family-based care (i.e. semi-independent living or residential care) had higher internalising and externalising problems and higher levels of anxiety and depression than those in family-based care (adoption, fostering or kinship).

A comparative review of prior literature, supplemented by interviews with psychologists, academics, social workers, and practitioners [19], concluded that adopted children in England exhibit more favourable mental health outcomes than those residing with special guardians or in foster care. These advantages are posited to facilitate smoother transitions into higher education, employment, and long‑term partnerships. Nonetheless, the continued provision of targeted support remains an important consideration, given the persistent uncertainty regarding the long‑term developmental effects of adversity experienced prior to entering care. Research with parents has presented a different perspective, demonstrating that children’s mental health difficulties remain elevated after adoption [20], and highlighting evidence of secondary trauma, long-term effects of child adversity, and children returning to care [21]. Research commissioned by the Parents of Traumatised Adopted Children [22] also described parents as being overwhelmed by a cluster of crises that could include mental health difficulties, self-harm and suicide, as well as school exclusion, substance misuse and contact with the criminal justice system. These crises have been linked back to early-life adversities [20, 23], yet families have also been critical of a lack of understanding or validation by services of their children’s needs, as well as limited professional support.

### The present study

This study is the first large-scale population study of the mental health of children in different living arrangements to include children who were in care and subsequently adopted. The research draws on data from a large national survey of secondary school children in mainstream schools across Wales to compare the mental health and wellbeing of children in adoptive families, kinship care, foster care and the general population. The adoptive sub-group included children who were previously in care but living in a private home with their adoptive parents at the time of the survey. The kinship group included those who were living with family members but not parents, with this potentially a formal care placement or informal arrangement, while the foster care sub-group included non-relative foster families. Finally, the general population group included children who were living with one or more of their parents. This included children who had previously spent time with other family members or in care and subsequently reunified (see Additional File 1). We hypothesised that: (1) children in the general population would have better mental health and wellbeing than those who were currently in care, or who had been in care and then adopted; and that (2) the mental health and wellbeing of adopted children would compare favourably to those who remained in (non-relative) foster care or kinship care.

## Method

### Design

The School Health Research Network (SHRN) is a strategic partnership between Cardiff University, the Welsh Government, and Public Health Wales that aims to improve children’s health and wellbeing in Wales, UK. Central to this is the Student Health and Wellbeing Survey, a large-scale study of children in Wales that has run every two years since 2017^1^. The survey collects data on areas including mental health, wellbeing and family, and was originally developed from the international Health Behaviour in School-aged Children (HBSC) survey, supported by the World Health Organization. A data resource profile [24] details the development and facilitation of the survey in full, including the core themes included in each wave, and sections that have been routed to a proportion of all respondents.

The most recent survey in 2023 was completed by 129,761 children aged 11–16 years. The children were from 202 of the 205 maintained secondary schools in Wales (95%) and included representation from all 22 local authorities. Previous iterations of the survey have enabled analyses of wellbeing depending on living arrangements, but the 2023 survey included adoption as a response option for the first time. This enabled the analysis of this group alongside non-relative foster care, kinship care and residential care (where children live in a shared home under the supervision of specialised staff), as well as children in the general population.

### Procedure and ethical considerations

Consent to participate in data collection was sought at three levels: school, parent/carer and child. All schools were invited to participate, with school-level consent obtained at survey registration. Parents and carers were informed about the survey through at least two methods of communication (i.e. letter, email, text message or web-based apps) and given the opportunity to withdraw their child via opt-out consent. Child assent was obtained at the beginning of the survey that was completed online and in class time. Participating children were informed that their participation was voluntary, that they could choose not to complete any items by selecting ‘I do not want to answer’ (an available response option for all questions except school year), and that they could stop the survey at any time. Ethical approval was obtained from Cardiff University School of Social Sciences Research Ethics Committee (SREC 431).

### Measures

#### Living arrangements

The survey asked children about the people that they were living with (*All families are different (for example, not everyone lives with both their parents; sometimes people live with just one parent, or they have two homes, or live with two families) and we would like to know about yours. Please answer this question for the home where you live all or most of the time and tick the ADULTS who live there*). The question had 13 response options with children able to select as many as were applicable to them. Four options (mother, father, mother’s partner and father’s partner) related to parents, and three options concerned other kin (aunt or uncle, grandparents, and adult brothers and/or sisters). Two response options related to currently living in the care of local authority (foster parents and residential care or children’s home), and one option was included for those who were in care and subsequently adopted (adoptive parent). Children could also select independent living or living with someone else (such as a friend), and there was also a refusal to answer option.

#### Mental health difficulties

The Strengths and Difficulties Questionnaire (SDQ) developed by Goodman [25, 26] is a widely used behavioural screening tool designed to assess psychosocial functioning in children and adolescents aged 2 to 17 years. The instrument comprises 25 items which are divided into five subscales: emotional, peer, conduct, hyperactivity difficulties, and prosocial behaviour. Items are rated on a three-point scale (0 = Not True, 1 = Somewhat True, 2 = Certainly True), with subscale scores ranging from 0 to 10. The SDQ data in SHRN demonstrates good internal consistency for total difficulties (α = 0.85), externalising (0.80), internalising (0.78) and prosocial behaviour (0.70).

#### Wellbeing

The Short Warwick–Edinburgh Mental Wellbeing Scale (SWEMWBS) is a psychometrically robust instrument designed to assess mental wellbeing in the general population. Developed as a shortened version of the original 14-item WEMWBS measure [27], the SWEMWBS comprises seven positively worded items that focus primarily on psychological functioning rather than emotional states. Each item is rated on a five-point scale ranging from “None of the time” (score = 1) to “All of the time” (score = 5), and total scores range from 7 to 35. The internal consistency estimate was good (α = 0.83). Anthony et al. [7] also found measurement invariance for care-experienced children compared to the general population, indicating the suitability of the measure for comparative profiling.

#### Depressive symptoms

The Short Mood and Feelings Questionnaire (SMFQ) is a widely used screening tool for depressive symptoms in children and adolescents. Originally developed by Angold et al. [28], the SMFQ is a 13-item unidimensional measure derived from the longer 33-item Mood and Feelings Questionnaire. Each item is rated on a three-point scale: “Not true” (0), “Sometimes true” (1), and “True” (2), yielding a total score ranging from 0 to 26. Higher scores indicate greater severity of depressive symptoms. While a score of 12 or above is often used as a threshold suggestive of clinically significant depression, there is no universally defined cut-off as optimal thresholds may vary depending on the population and context of use. The SMFQ has demonstrated strong psychometric properties and has been validated for use across developmental stages and demographic groups [29]. The SMFQ section of the Student Health and Wellbeing Survey is routed to half the participating schools, resulting in approximately half of the children receiving these questions. The internal consistency of the SMFQ measure was excellent for the SHRN dataset (α = 0.93).

#### Socio-demographic characteristics

Children were asked about their gender with options for identifying as a ‘boy’ (1), ‘girl’ (2) or ‘neither word describes me’ (3). A variable on sex at birth with three response options (including a refusal to answer) was included for the purpose of imputing missing data and transformed into a binary variable (male = 0; female = 1). An ethnicity variable in the student survey with 14 response options was collapsed into a revised variable with five groups (Asian or Asian British; Black or Black British; Mixed or Multiple Ethnicities; Other Ethnic Groups; White). A question on school year was used as a proxy variable for age due to there being no missing data.

Socioeconomic status was assessed through the Family Affluence Scale (FAS), a widely used, child-reported measure of material wealth among children and adolescents [30]. The FAS included six items that capture tangible aspects of family wealth: car ownership, bedroom occupancy (i.e., whether the child has their own bedroom), number of family holidays outside of Wales, number of computers/tablets in the household, dishwasher ownership, and number of bathrooms. Each item was scored and summed to produce a composite score. The FAS has demonstrated good construct and criterion validity across multiple countries and cultural contexts, though internal consistency was low (α = 0.59) for the SHRN dataset. The scale also correlates moderately with parental income and employment status [30], with it particularly effective at distinguishing between low- and high-income households.

### Data analysis

**Fig. 1:**
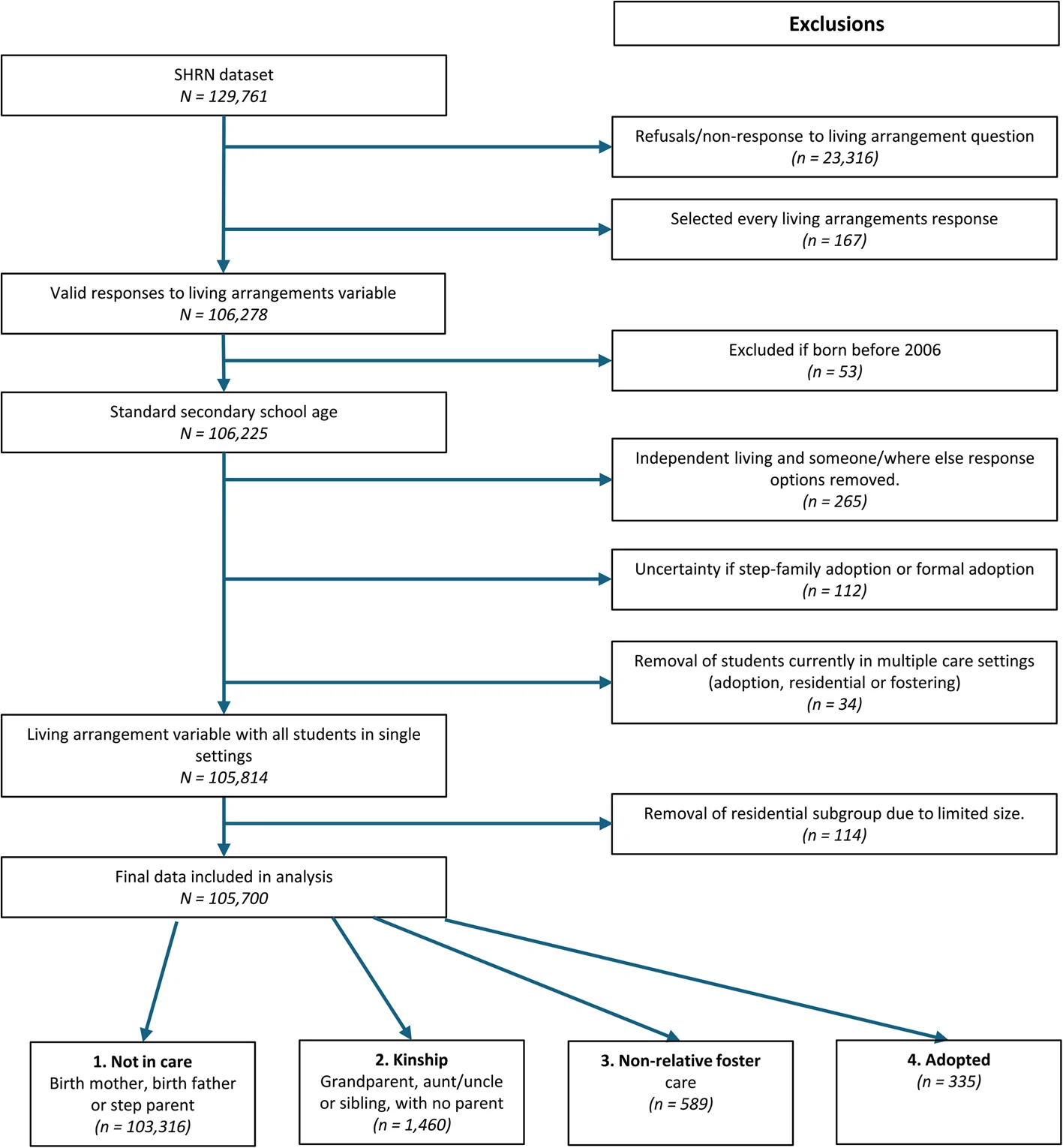
Flow diagram of the process followed for excluding invalid cases. The diagram also details the development of a current living arrangement variable with every child living in a single setting

The original living arrangements variable was reduced to a revised variable with four sub-groups: adoptive, kinship, non-relative foster care, and general population. Three living arrangement subgroups were excluded including the residential care group which was too small to include, the independent living subgroup and the living with someone else group. Children who selected every option or refused to answer were also discounted. The large number of response options resulted in inconsistencies that needed resolution. This was achieved through reasoning the true living arrangements or removing cases where this was not possible. Figure 1 summarises the development of the living arrangements variable, and the specific decisions are explained in Additional File 2.

The data were analysed in R using the mice, miceadds, mitml and lme4 packages for multiple imputation and multi-level modelling. Missing data due to non-response by children was low in the final dataset (3.3%). This included no missing data in the living arrangements variable (non-response cases excluded), school year variable (required response) or school identification number (administrative data). Missing data did however exceed 5% on eight of the SDQ items. The *p*-value for the Little’s MCAR test was greater than 0.999, indicating that the data was missing completely at random.

Multiple imputation was used to estimate data that was missing due to non-response. This did not include the structurally missing data for the SMFQ measure where children had not received the questions due to routing. The imputation resulted in five completed datasets where the data provided by the children remains the same, but where each missing value was estimated multiple times based on correlations (Additional File 3). There was multi-collinearity (0.95) between the sex at birth and gender variables that had 0.93% and 0.91% missing data respectively, though this was expected given the close conceptual relationship between sex and gender. The decision to create five completed datasets is in line with guidance that the number of imputations should be greater than the percentage of missing data [31].

Multi-level linear regression modelling was chosen to account for differences in living arrangements. The general population group was selected as the reference group (0) due to it being the largest group, and dummy variables were developed for the kinship, adoption and fostering groups. Total scores were summed for the different measures including the Family Affluence Scale, SDQ, SMFQ and SWEMWBS. Individual models were run for the respective mental health difficulties, wellbeing and depressive symptoms. Each of the models included demographics as covariates, with the lowest year group and minimum affluence score selected as referent groups. Referent groups for the categorical variables were selected based on the largest subgroup (i.e. girls and white ethnicity). The models also included school as a cluster variable to discount the effect of similarities because of children attending the same school. Adjusting the model results to account for the non-independence of school-level data enabled a greater focus on differences between children in the different living arrangements. The models were run for each of the five imputed datasets and then pooled into a single set of results. The analysis was repeated for the non-imputed data (i.e. complete cases only), and with the residential subgroup included, for the purpose of comparison.

## Results

### Descriptive analysis

The 2022/23 Student Health and Wellbeing Survey was completed by 129,761 children in 202 schools in Wales. Following the data cleaning process, 24,061 cases were excluded, largely due to non-response to the living arrangements question (*n* = 23,316). Cases were also removed due to children selecting every response option to the living arrangements question (*n* = 167), reporting being in multiple forms of care (*n* = 34), or selecting that they lived independently or were ‘living somewhere else’ (*n* = 265), as well as uncertainty over if cases were formal adoption or step-family adoptions (*n* = 112). The residential care subgroup was removed as it was too small to include (*n* = 114). In addition, 53 cases were excluded due to the children being above the standard secondary school age.

After data cleaning the final sample size included 105,700 respondents, including 103,316 in the general population group. A total of 2,384 care-experienced children were in three settings: kinship care (*n* = 1,460), non-relative foster care (*n* = 589) and adoptive families (*n* = 335). Table 1 reports the summary statistics of the final sample, with the ethnicity, sex and gender subgroups listed alphabetically, consistent with APA good practice [32]. Slightly more children were female at birth (51.9%) than male. More identified as girls (50.6%), compared to 48.1% boys, while 1.3% answered that neither word described them. More children in the care-experienced groups identified as neither a girl or boy; this was highest for the adopted group (7.0%) compared to fostering (4.0%) and kinship placements (2.6%).

**Table 1:** Sample characteristics for the different living arrangements groups in the sample

	General population		Kinship		Foster		Adoption		Total	
	N	%	N	%	N	%	N	%	N	%
Living arrangements	103,316	97.6	1460	1.4	589	0.6	335	0.3	105,700	100
Gender										
Boy	49,277	48.1	678	47.1	275	47.3	151	46.2	50,381	48.1
Girl	51,824	50.6	723	50.2	284	48.8	153	46.8	52,984	50.6
Neither word describes me	1294	1.3	38	2.6	23	4.0	23	7.0%	1378	1.3
Sex at birth										
Female	53,157	51.9	746	52.3	293	51.6	156	48.9	54,392	51.9
Male	49,244	48.1	680	47.7	275	48.4	163	51.1	50,362	48.1
School year										
7	20,880	20.2	312	21.4	120	20.4	64	19.1	21,376	20.2
8	21,932	21.2	260	17.8	132	22.4	71	21.2	22,395	21.2
9	21,866	21.2	312	21.4	117	19.9	73	21.8	22,368	21.2
10	19,884	19.2	281	19.2	107	18.2	62	18.5	20,334	19.1
11	18,754	18.2	295	20.2	113	19.2	65	19.4	19,227	18.2
Ethnicity										
Asian/Asian British	4630	4.7	43	3.2	8	1.5	11	3.6	1864	1.8
Black/Black British	1810	1.8	32	2.4	11	2.0	11	3.6	1864	1.8
Mixed/multiple ethnic groups	3284	3.3	63	4.7	25	4.6	12	3.9	4692	4.6
Other	1989	2.0	18	1.3	19	3.5	6	2.0	2032	2.0
White	87,341	88.2	1198	88.5	478	88.4	266	86.9	89,283	88.2

Table 2 includes the group-level means and standard deviations for the measures and demographics. Affluence was greater in the adoptive group (*M* = 10.0; *SD* = 2.4) than all other groups, including children who were in the general population (*M* = 9.3; *SD* = 2.4). The general population group had the best outcomes for all measures, including the SMFQ (*M* = 7.6; *SD* = 6.7), the SDQ difficulties scale (*M* = 14.4; *SD* = 7.0), and the SWEMWEBS wellbeing measure (*M* = 23.6; *SD* = 5.4). The adoption subgroup scored higher on the SDQ total difficulties scale (*M* = 18.7; *SD* = 7.2) than the kinship and fostering groups. The adopted group also scored highest on the SMFQ for depressive symptoms (*M* = 11.5; *SD* = 8.2), and lowest on the SWEMWEBS scale indicating poor wellbeing (*M* = 20.9 *SD* = 6.5), though there was little difference in SDQ prosocial behaviours between the three care-experienced groups.

**Table 2:** Group means and standard deviations for the different living arrangements

	General population			Kinship			Foster			Adoption			Total		
	N	Mean	SD	N	Mean	SD	N	Mean	SD	N	Mean	SD	N	Mean	SD
Affluence	97,890	9.3	2.4	1362	8.6	2.4	530	9.4	2.3	312	10.0	2.4	100,094	9.3	2.4
Short moods and feelings*	46,555	7.6	6.7	655	9.9	7.6	251	8.9	7.9	133	11.5	8.2	47,594	7.7	6.8
Wellbeing	95,499	23.6	5.4	1299	21.6	6.0	506	22.3	6.3	297	20.9	6.5	97,601	23.6	5.5
Strengths and difficulties															
SDQ difficulties	89,945	14.4	7.0	1190	17.5	6.9	573	16.3	7.2	265	18.7	7.2	91,873	14.5	7.1
SDQ internalising	92,619	6.6	4.0	1237	8.0	4.2	493	7.5	4.3	282	8.5	4.2	94,631	6.7	4.0
SDQ externalising	92,989	7.8	4.2	1230	9.6	4.0	500	8.8	4.0	280	10.2	4.1	94,999	7.9	4.2
SDQ prosocial	95,792	7.2	2.1	1300	6.7	2.5	518	6.8	2.6	301	6.7	2.7	97,911	7.2	2.1

^*^The Short Moods and Feeling Questionnaire were routed to only schools in the mental health cohort

### Multi-level models

Table 3 presents the unstandardised results of the different multi-level regression models, including confidence intervals, with standardised and full *p*-values included in Additional File 4. Children in the general population group had fewer difficulties than the care-experienced groups for all SDQ measures, including the total difficulties score (*b* = 13.28), internalising (*b* = 6.91) and externalising difficulties (*b* = 6.38). They also scored lower on the SMFQ measure for depressive symptoms (*b* = 4.45), while higher scores on the SWEMWEBS scale (*b* = 18.43) indicated better wellbeing.

**Table 3:** Multi-level regression model results

	Estimate	SE	***t***	***p***	LCI	UCI
SDQ total difficulties						
(Intercept)	13.28***	0.17	77.52	0.00	12.94	13.61
Kinship	1.91***	0.19	10.30	0.00	1.55	2.27
Fostering	1.00***	0.29	3.44	0.00	0.43	1.57
Adopted	3.53***	0.38	9.17	0.00	2.78	4.29
Boy	−2.66***	0.04	−60.73	0.00	−2.75	−2.58
Neither (gender)	3.70***	0.19	18.97	0.00	3.32	4.08
School year	0.57***	0.02	36.26	0.00	0.54	0.60
Asian/Asian British	−2.14***	0.11	−18.93	0.00	−2.37	−1.92
Mixed or multiple	0.27*	0.13	2.12	0.03	0.02	0.52
Other (ethnicity)	−0.98***	0.17	−5.88	0.00	−1.31	−0.65
Black/Black British	−2.62***	0.17	−15.67	0.00	−2.95	−2.29
Affluence	−0.32***	0.01	−35.47	0.00	−0.34	−0.31
ICC:	0.009					
SDQ internalising difficulties						
(Intercept)	6.91***	0.09	73.91	0.00	6.72	7.09
Kinship	0.76***	0.10	7.44	0.00	0.56	0.96
Fostering	0.48***	0.16	3.01	0.00	0.17	0.80
Adopted	1.53***	0.21	7.22	0.00	1.12	1.95
Boy	−2.05***	0.02	−84.75	0.00	−2.09	−2.00
Neither (gender)	2.04***	0.11	18.99	0.00	1.83	2.25
School year	0.28***	0.01	32.12	0.00	0.26	0.29
Asian/Asian British	−0.73***	0.06	−11.87	0.00	−0.85	−0.61
Mixed or multiple	−0.03	0.07	−0.47	0.64	−0.17	0.11
Other (ethnicity)	−0.42***	0.09	−4.64	0.00	−0.60	−0.24
Black/Black British	−1.18***	0.09	−12.93	0.00	−1.36	−1.01
Affluence	−0.21***	0.01	−41.76	0.00	−0.22	−0.20
ICC:	0.007					
SDQ externalising difficulties						
(Intercept)	6.38***	0.10	62.49	0.00	6.18	6.58
Kinship	1.15***	0.11	10.36	0.00	0.93	1.37
Fostering	0.52***	0.17	2.96	0.00	0.17	0.86
Adopted	2.00***	0.23	8.66	0.00	1.54	2.45
Boy	−0.62***	0.03	−23.44	0.00	−0.67	−0.56
Neither (gender)	1.66***	0.12	14.35	0.00	1.44	1.89
School year	0.29***	0.01	30.96	0.00	0.27	0.31
Asian/Asian British	−1.43***	0.07	−21.03	0.00	−1.56	−1.29
Mixed or multiple	0.30***	0.08	3.98	0.00	0.15	0.45
Other (ethnicity)	−0.57***	0.10	−5.73	0.00	−0.76	−0.37
Black/Black British	−1.44***	0.10	−14.42	0.00	−1.64	−1.25
Affluence	−0.11***	0.01	−21.02	0.00	−0.13	−0.10
ICC:	0.008					
SDQ prosocial						
(Intercept)	8.36***	0.06	143.32	0.00	8.25	8.48
Kinship	−0.56***	0.06	−8.88	0.00	−0.68	−0.43
Fostering	−0.54***	0.10	−5.50	0.00	−0.73	−0.35
Adopted	−0.65***	0.13	−4.98	0.00	−0.90	−0.39
Boy	−0.96***	0.01	−65.34	0.00	−0.99	−0.94
Neither (gender)	−1.31***	0.07	−20.02	0.00	−1.44	−1.18
School year	−0.19***	0.01	−35.31	0.00	−0.20	−0.18
Asian/Asian British	−0.15***	0.04	−4.06	0.00	−0.23	−0.08
Mixed or multiple	−0.22***	0.04	−5.08	0.00	−0.30	−0.13
Other (ethnicity)	−0.15**	0.05	−2.75	0.01	−0.26	−0.04
Black/Black British	−0.13*	0.06	−2.18	0.03	−0.25	−0.01
Affluence	0.07***	0.00	23.08	0.00	0.07	0.08
ICC:	0.012					
Wellbeing						
(Intercept)	18.43***	0.14	129.18	0.00	18.15	18.71
Kinship	−1.83***	0.16	−11.77	0.00	−2.14	−1.53
Fostering	−1.76***	0.24	−7.23	0.00	−2.24	−1.29
Adopted	−3.12***	0.32	−9.63	0.00	−3.75	−2.48
Boy	2.31***	0.04	62.29	0.00	2.23	2.38
Neither (gender)	−3.02***	0.17	−18.28	0.00	−3.34	−2.69
School year	−0.10***	0.01	−7.65	0.00	−0.13	−0.07
Asian/Asian British	0.45***	0.10	4.46	0.00	0.25	0.64
Mixed or multiple	−0.10	0.11	−0.97	0.33	−0.31	0.11
Other (ethnicity)	−0.06	0.14	−0.42	0.68	−0.33	0.21
Black/Black British	1.60***	0.15	10.33	0.00	1.29	1.90
Affluence	0.46***	0.01	60.45	0.00	0.45	0.48
ICC:	0.007					
SMFQ						
(Intercept)	4.45***	0.29	15.15	0.00	3.87	5.02
Kinship	0.91***	0.12	7.52	0.00	0.67	1.14
Fostering	0.51**	0.19	2.68	0.01	0.14	0.88
Adopted	1.48***	0.25	5.92	0.00	0.99	1.97
Boy	−1.93***	0.03	−67.50	0.00	−1.98	−1.87
Neither (gender)	1.90***	0.13	15.08	0.00	1.66	2.15
School year	0.17***	0.01	17.01	0.00	0.15	0.19
Asian/Asian British	−0.28***	0.07	−3.77	0.00	−0.42	−0.13
Mixed or multiple	0.23***	0.08	2.91	0.00	0.08	0.39
Other (ethnicity)	−0.05	0.11	−0.45	0.65	−0.26	0.16
Black/Black British	−0.67***	0.11	−6.17	0.00	−0.88	−0.46
Affluence	−0.15***	0.01	−25.15	0.00	−0.16	−0.14
ICC:	0.010					

The intercept group is the reference category that reflects children who are in the general population, girls and white, each selected as the largest subgroup of the respective variable. The remaining living arrangement, gender and ethnicity subgroups are estimated respective to this. The reference group also reflects children in Year 7 and minimum affluence. The estimate for school year and affluence reflects the change in outcomes for each subsequent year or affluence point

Asterisks indicate statistical significance of the estimated coefficients. * *p* < 0.05, ** *p* < 0.01, *** *p* < 0.00

Higher ICC (Intraclass Correlation Coefficient) values indicate greater variance between schools, rather than within schools

All results for the care-experienced subgroups are relative to the general population as the reference group. The adopted subgroup scored higher on the SDQ total difficulties scale (*b* = 3.53) than the general population group, but they also had greater difficulties than the kinship and fostering groups (*b* = 1.91 and 1.00 respectively). The results were similar for the internalising and externalising scores with the fostering group having the best outcomes, followed by the kinship and adoption subgroups. There was less difference for the inverse prosocial subscale with the adopted group having slightly lower prosocial behaviour (*b* = −0.65) than the fostering (*b* = −0.54) and kinship groups (*b* = −0.56). The adoption group also had the lowest scores on the SWEMWEBS wellbeing scale (*b* = −3.12) compared to the kinship (*b* = −1.83) and fostering groups (*b* = −1.76), and for the SMFQ scale for depressive symptoms (*b* = 1.48, compared to 0.91 and 0.51). All *p*-values for living arrangements were statistically significant (*p* < 0.01). The results of the list-wise analysis reported in Additional File 5 indicate some differences in magnitude to those for the imputed data, but the substantive results are the same.

Several demographic covariates showed significant associations with the outcomes in the three models. Most associations were small, though children who did not identify as either boys or girls reported particularly negative outcomes. This included higher total SDQ difficulties (*b* = *3.70*) compared to boys (*b* = *−2.*66) with girls as the reference group, and lower wellbeing (*b* = *−3.02*) than boys (*b* = *2.31*), though the subgroup is limited in size. Based on school year as a proxy, mental health symptoms increased and wellbeing was lower as age increased. More affluent children had better outcomes for all measures. Black children reported the most positive outcomes of the different ethnicity groups for most measures, though White children reported higher prosocial behaviour. Probability values were < 0.01 for all gender, school year and affluence covariates. A minority of ethnicity covariate probability values exceeded this, potentially due to the small subgroup sizes.

The models included school as a cluster variable to account for the non-independence of school-level data. The level of clustering was assessed through the Intraclass Correlation Coefficient (ICC). Clustering was weak in all models (e.g. 0.007 in both the SDQ internalising difficulties and the SWEMWEBS wellbeing scale). This indicated that the nesting of children within schools explained little of the total variance, with the majority located at the individual level.

## Discussion

There have been a limited number of large-scale studies comparing the mental health of children in care with those in the general population [5, 6, 8, 23], and there is a notable gap in population-based research involving children who were previously in care and subsequently adopted [18]. We drew on population-level data from a nationally representative study of secondary school pupils in Wales to examine the mental health and wellbeing profiles of children across different living arrangements. We hypothesised that: (1) children in the general population would exhibit better mental health and wellbeing outcomes than the care-experienced groups; and (2) adopted children would have more favourable outcomes than children who remained in local authority care.

The first hypothesis was supported, with the general population having better outcomes across all domains, when controlling for covariates including gender, school year, ethnicity and affluence. School year was used as a proxy for age. Mental health symptoms increased and wellbeing declined with advancing school year, consistent with the broader social and developmental changes associated with adolescence [33]. Contrary to our second hypothesis, adopted children exhibited the poorest outcomes of the care-experienced groups across multiple domains including elevated total difficulties on the Strengths and Difficulties Questionnaire (SDQ), higher internalising and externalising symptoms, increased depressive symptoms, and lower overall wellbeing scores. Differences in prosocial skills were less pronounced, though adopted children still scored slightly lower than other care-experienced groups. Overall, these findings challenge prevailing assumptions that adoption, as a permanent and stable placement, typically leads to improved psychological outcomes compared to remaining in care.

While adoptive placements generally function well for many families [34], the relatively poor mental health and wellbeing identified in the present study may reflect a constellation of pre- and post-natal risk. These risk factors, often measured using categorical indices such as Adverse Childhood Experiences (ACEs) checklists, have been positively associated with both internalising and externalising difficulties in adopted children [20, 23], and Engler et al. [14] has demonstrated that specific forms of maltreatment are differentially associated with mental health disorders. These patterns may help explain the elevated symptomatology observed in adopted children, who have often experienced multiple forms of maltreatment. While this paper specifically considered living arrangements and placement type, other aspects of time in care have been identified as potentially important contextual factors. A recent UK systematic review and meta-analysis [35] found that that care experienced children with unstable placements were more than twice as likely to experience mental health problems compared to children in stable placements. Additionally, children who enter care at an older age are more likely to experience placement instability and developmental delays [36], potentially compounding mental health difficulties. It is also likely that the impacts of both the pre-care adversity and in-care factors are cumulative, with a narrative systematic review [17] assessing how experience of maltreatment, being in care, placement type, instability, and age at placement can all contribute to future behaviour.

The annual Adoption UK Barometer surveys [37] have consistently highlighted that post-adoption experiences can be complex, with evidence of disrupted attachments, trauma-related behaviours and a lack of post-adoption support. There is a legal requirement for children in fostering or residential care to have annual health assessments that inform referrals and the accessing of services [38], with Palacios contrasting this with the right of adoptive families to request assessments for services that often have limited funding. Adopted children, like other care experienced children, frequently receive inconsistent, fragmented or minimal life story work relating to their earlier lives, despite the existence of policy and practice guides [39] recommending this. Uneven practice in the use and quality of life story work may have consequences for a child’s ability to construct and interpret their experiences [40]. Research with the adoptive families of traumatised children and young adults has also highlighted the secondary trauma of parents [21] and their challenges in accessing and engaging with good quality mental health services [41]. For some this has resulted in a clustering of crises relating to pre-care experiences, and they have been left to navigate these challenges alone [22].

Our findings contrast with other work that has identified better outcomes for adoptive children compared to those who remained in care [19], and there is a clear need to build an evidence base for what works in supporting adopted children and their families. Woolgar et al. [41] highlighted the importance of developing a shared understanding of the psychological needs of adoptive children; this could include a renewed research focus on children, parents, practitioners and professionals (in health and education) to consider the practice and policy change that is needed to improve outcomes for adopted children.

### Strengths and limitations

To our knowledge, this study represents the first large-scale analysis of mental health and wellbeing outcomes for adopted children using nationally representative UK data. The inclusion of children aged 11–16 corresponds with the full span of mainstream secondary education, and the study treats school year as a continuum while controlling for developmental differences. The resulting sample size enabled the inclusion of three groups of care-experienced children (adopted, kinship care and non-relative foster placements) alongside a general population group. Non-response was low across the whole dataset, and the data missing completely at random. While the use of population level data lends robustness to the conclusions, the nature of large datasets necessitates cautious interpretation of confidence intervals.

Limitations include the reliance on youth self report. The living arrangements variable was particularly complex, comprising 13 response options, and interpreting the actual circumstances of a minority of participants proved challenging. This was especially evident among care experienced children, with some selecting that they were currently in adoptive families, fostering and residential care. This combination may reflect movement between placement types or uncertainty about their primary residence. Although a systematic approach was applied to infer the most plausible arrangement where possible, and to exclude cases that remained ambiguous, there remains a risk of misclassification bias. Another limitation concerns the exclusion of children who were in residential care, and studies [5, 7] have demonstrated particularly poor outcomes for this population. Children in residential care were not included in the paper due to the small sample size, but Additional File 6 includes this group in the models. Their outcomes were poor compared to the fostering and kinship groups, but more positive than the adoptive group for the SDQ total difficulties and the SMFQ scales.

The Student Health and Wellbeing Survey is completed by school children (aged 11 to 16) in mainstream education. While most adopted children attend mainstream schools, an estimated 40% of children currently in care in England attend alternative provision [42]. With alternative provision aimed at those with additional support needs, our findings may therefore reflect the absence of children in kinship or foster care with greater needs from mainstream schooling. The research was also limited by the indicators included in the survey. For example, children with other forms of contact with social services (e.g. assessed as not in need, or as in need but not in care) also have an increased likelihood of poor mental health [6], but information about referrals within the health system was not assessed with this dataset. Furthermore, we have highlighted several contextual factors beyond living arrangements (e.g. age entering care, time in care or placement instability) that may cause variation in outcomes for children who are adopted, in fostering or kinship care. Additional factors specific to the outcomes of adopted children include openness within the adoptive family [43] and contact with their birth family [44]. However, with the Student Health and Wellbeing Survey only collecting data on living arrangements, it is not suitable for this more nuanced investigation of differences within the care-experienced population. There is clear potential for future research with administrative linked datasets. Acknowledging the inherent complexity underlying the origins of mental health problems and lower wellbeing in care-experienced populations highlights the need for longitudinal studies that can track psychosocial interactions over time and in concert with placement changes.

### Implications for policy and practice

The disparities identified in this study reinforce the urgent need for evidence-based mental health support for care-experienced children, including adopted children. Access to support services remains contentious, including an apparent reluctance to use standard protocols and diagnostic assessments to formulate a child’s support needs (e.g. [45]). One important example is the Adoption and Special Guardianship Support Fund (ASGSF), a government scheme that provides funding for therapeutic services for families living in England. Independent evaluations of the fund [46–48] coupled with urgent calls for commissioners to adopt an evidence-based approach to decision making [41, 45] should give pause for thought; indeed, over and again evidence highlights that care-experienced children should have access to the same transdiagnostic assessments and evidence based interventions as other children. Overall, our findings add to a growing literature that raise critical questions about how limited government resources and approaches to screening can equitably meet the mental health and wellbeing needs of care-experienced children [18, 49].

## Conclusion

This study contributes to a growing body of evidence on the mental health needs of care-experienced children and adolescents. The greater challenges reported by adopted children highlight the need for nuanced approaches that extend beyond placement stability. Moreover, while schools increasingly prioritise wellbeing in their communities, care-experienced pupils remain underserved by such initiatives [50]. This gap presents a critical opportunity for educational settings to play a more active role in supporting vulnerable children. Future research should explore the interplay of intergenerational transmission of risk and systemic factors, while also prioritising longitudinal designs to capture developmental trajectories. Policy and practice must evolve to ensure that all care-experienced children, including those who have left care through adoption, receive the support they need to thrive.

## Supplementary Information

Additional file 1.

Additional file 2.

Additional file 3.

Additional file 4.

Additional file 5.

Additional file 6.

## Data Availability

Data from the Student Health and Well-being (SHW) surveys are available for research purposes. Applicants will need to complete a data access request and re-turn a signed data use protocol. Data requests will be reviewed, and a decision regarding data access made within 6 weeks. Provisioning of data will commence following confirmation of approval. Please contact SHRN at Cardiff University for further details (SHRN@cardiff.ac.uk).

## Abbreviations

FAS: Family Affluence Scale
SDQ: Strengths and Difficulties Questionnaire
SMFQ: Short Moods and Feelings Questionnaire
SWEMWBS: Short Warwick–Edinburgh Mental Wellbeing Scale

## Footnotes

1 https://publichealthwales.shinyapps.io/SHRN_Dashboard/.

## References

[1] Tarren-Sweeney M. The mental health of children in out-of-home care. Curr Opin Psychiatry. 2008;21(4):345. 10.1097/YCO.0b013e32830321fa. 18520738 10.1097/YCO.0b013e32830321fa

[2] Department for Education. Family Routes study: making decisions about their children’s care. Department for Education; 2025. Report No.

[3] Rutter M. Children in substitute care: some conceptual considerations and research implications. Child Youth Serv Rev. 2000;22(9–10):685–703. 10.1016/S0190-7409(00)00116-X.

[4] McLaughlin KA, Colich NL, Rodman AM, Weissman DG. Mechanisms linking childhood trauma exposure and psychopathology: a transdiagnostic model of risk and resilience. BMC Med. 2020;18(1):96. 10.1186/s12916-020-01561-6. 32238167 10.1186/s12916-020-01561-6 PMC7110745

[5] Ford T, Vostanis P, Meltzer H, Goodman R. Psychiatric disorder among British children looked after by local authorities: comparison with children living in private households. Br J Psychiatry. 2007;190(4):319–25. 10.1192/bjp.bp.106.025023. 17401038 10.1192/bjp.bp.106.025023

[6] McKenna S, O’Reilly D, Maguire A. The mental health of all children in contact with social services: a population-wide record-linkage study in Northern Ireland. Epidemiol Psychiatr Sci. 2023;32:e35. 10.1017/S2045796023000276. 37190768 10.1017/S2045796023000276 PMC10227534

[7] Anthony R, Moore G, Page N, Hewitt G, Murphy S, Melendez-Torres GJ. Measurement invariance of the short Warwick-Edinburgh Mental Wellbeing Scale and latent mean differences (SWEMWBS) in young people by current care status. Qual Life Res. 2022;31(1):205–13. 10.1007/s11136-021-02896-0. 34050443 10.1007/s11136-021-02896-0 PMC8800901

[8] Long SJ, Evans RE, Fletcher A, Hewitt G, Murphy S, Young H, et al. Comparison of substance use, subjective well-being and interpersonal relationships among young people in foster care and private households: a cross sectional analysis of the School Health Research Network survey in Wales. BMJ Open. 2017;7(2):e014198. 10.1136/bmjopen-2016-014198. PubMed PMID: 28219960. 28219960 10.1136/bmjopen-2016-014198 PMC5337680

[9] Evans R, White J, Turley R, Slater T, Morgan H, Strange H, et al. Comparison of suicidal ideation, suicide attempt and suicide in children and young people in care and non-care populations: systematic review and meta-analysis of prevalence. Child Youth Serv Rev. 2017;82:122–9. 10.1016/j.childyouth.2017.09.020.

[10] Seker S, Boonmann C, Gerger H, Jäggi L, d’Huart D, Schmeck K, et al. Mental disorders among adults formerly in out-of-home care: a systematic review and meta-analysis of longitudinal studies. Eur Child Adolesc Psychiatry. 2022;31(12):1963–82. 10.1007/s00787-021-01828-0. 34169369 10.1007/s00787-021-01828-0 PMC9663399

[11] Bronsard G, Alessandrini M, Fond G, Loundou A, Auquier P, Tordjman S, et al. The prevalence of mental disorders among children and adolescents in the child welfare system: a systematic review and meta-analysis. Medicine Baltimore. 2016;95(7):e2622. 10.1097/MD.0000000000002622. 26886603 10.1097/MD.0000000000002622 PMC4998603

[12] Cummings A, Shelton K. The prevalence of mental health disorders amongst care-experienced young people in the UK: a systematic review. Child Youth Serv Rev. 2024;156:107367. 10.1016/j.childyouth.2023.107367.

[13] Dubois-Comtois K, Bussières EL, Cyr C, St-Onge J, Baudry C, Milot T, et al. Are children and adolescents in foster care at greater risk of mental health problems than their counterparts? A *meta*-analysis. Child Youth Serv Rev. 2021;127:106100. 10.1016/j.childyouth.2021.106100.

[14] Engler AD, Sarpong KO, Van Horne BS, Greeley CS, Keefe RJ. A systematic review of mental health disorders of children in foster care. Trauma Violence Abuse. 2022;23(1):255–64. 10.1177/1524838020941197. 32686611 10.1177/1524838020941197

[15] Winokur MA, Holtan A, Batchelder KE. Systematic review of kinship care effects on safety, permanency, and well-being outcomes. Res Soc Work Pract. 2018;28(1):19–32. 10.1177/1049731515620843.

[16] Lou Y, Taylor EP, Di Folco S. Resilience and resilience factors in children in residential care: a systematic review. Child Youth Serv Rev. 2018;89:83–92. 10.1016/j.childyouth.2018.04.010.

[17] Lee JJ, Holmes L. Longitudinal trajectories of behavioral problems among children in out-of-home care: a systematic review. Child Youth Serv Rev. 2021;127:106086. 10.1016/j.childyouth.2021.106086.

[18] Carter B, Shelton KH, Holmes LJ, Sprecher EA, Javed M, Macleod J, et al. The mental health and wellbeing of care-experienced young people during early and later adolescence. Clin Child Psychol Psychiatry. 2025;30(3):611–31. 10.1177/13591045251333028. 40257037 10.1177/13591045251333028 PMC12179413

[19] Clifford J, Hutchison E, Hulbert A, Kemp J, Turrell M. A comparative review of the value of different forms of permanence for children – Adoption, SGOs and Fostering. London: Sonnet Impact; 2022. Report No.

[20] Paine AL, Fahey K, Anthony RE, Shelton KH. Early adversity predicts adoptees’ enduring emotional and behavioral problems in childhood. Eur Child Adolesc Psychiatry. 2021;30(5):721–32. 10.1007/s00787-020-01553-0. 32468437 10.1007/s00787-020-01553-0 PMC8060221

[21] O’Marah R, Hodge S, Machin L. Caring for traumatized children: a systematic review exploring experiences of secondary trauma and compassion fatigue among adoptive and foster parents. Adop Q. 2025;0(0):1–31. 10.1080/10926755.2025.2557222.

[22] The Potato Group. Far, Far Beyond the Adoption Order. The Potato Group; 2025. Report No.

[23] Anthony RE, Paine AL, Shelton KH. Depression and anxiety symptoms of British adoptive parents: a prospective four-wave longitudinal study. Int J Environ Res Public Health. 2019;16(24):24. 10.3390/ijerph16245153. 10.3390/ijerph16245153 PMC6949987 31861100

[24] Page N, Liu S, Morgan K, Angel L, Ogada E, Roberts C, et al. Data resource profile: the school health research network (SHRN) student health and well-being (SHW) survey of 11–16-year-olds (2017–2023). Int J Epidemiol. 2024;53(6):dyae161. 10.1093/ije/dyae161. 39657065 10.1093/ije/dyae161 PMC11645470

[25] Goodman R. The Strengths and Difficulties Questionnaire: a research note. J Child Psychol Psychiatry. 1997;38(5):581–6. 10.1111/j.1469-7610.1997.tb01545.x. 9255702 10.1111/j.1469-7610.1997.tb01545.x

[26] Goodman R. Psychometric properties of the Strengths and Difficulties Questionnaire. J Am Acad Child Adolesc Psychiatry. 2001;40(11):1337–45. 10.1097/00004583-200111000-00015. 11699809 10.1097/00004583-200111000-00015

[27] Tennant R, Hiller L, Fishwick R, Platt S, Joseph S, Weich S, et al. The Warwick-Edinburgh mental well-being scale (WEMWBS): development and UK validation. Health Qual Life Outcomes. 2007;5:63. 10.1186/1477-7525-5-63. 18042300 10.1186/1477-7525-5-63 PMC2222612

[28] Angold A, Costello EJ, Messer SC, Pickles A. Development of a short questionnaire for use in epidemiological studies of depression in children and adolescents. Int J Methods Psychiatr Res. 1995;5(4):237–49.

[29] Schlechter P, Wilkinson PO, Ford TJ, Neufeld SAS. The Short Mood and Feelings Questionnaire from adolescence to emerging adulthood: measurement invariance across time and sex. Psychol Assess. 2023;35(5):405–18. 10.1037/pas0001222. 36951690 10.1037/pas0001222

[30] Currie C, Alemán Díaz AY, Bosáková L, de Looze M. The international Family Affluence Scale (FAS): charting 25 years of indicator development, evidence produced, and policy impact on adolescent health inequalities. SSM Popul Health. 2024;25:101599. 10.1016/j.ssmph.2023.101599. 38313871 10.1016/j.ssmph.2023.101599 PMC10835442

[31] Pedersen AB, Mikkelsen EM, Cronin-Fenton D, Kristensen NR, Pham TM, Pedersen L, et al. Missing data and multiple imputation in clinical epidemiological research. Clin Epidemiol. 2017;9:157–66. 10.2147/CLEP.S129785. PubMed PMID: 28352203; PubMed Central PMCID: PMC5358992. 28352203 10.2147/CLEP.S129785 PMC5358992

[32] American Psychological Association. Journal Article Reporting Standards: Race, Ethnicity, and Culture (JARS–REC), Table 1: Information Recommended for Inclusion in All Manuscripts. APA Style. 2023. Available from: https://apastyle.apa.org/jars/rec-table-1.pdf.

[33] Tolstrup JS, Larsen SR, Kelleher I, Cannon M, Lytken Larsen CV, Nordentoft M. Mental well-being in adolescence and eight years of follow-up for mental illness, risky behaviours, and mortality in 67,945 15-19-year-olds: a prospective cohort study. Lancet Reg Health Eur. 2025;58:101435. 10.1016/j.lanepe.2025.101435. PubMed PMID: 40989561; PubMed Central PMCID: PMC12451365. 40989561 10.1016/j.lanepe.2025.101435 PMC12451365

[34] Selwyn J, Wijedasa D, Meakings S. Beyond the Adoption Order: challenges, interventions and adoption disruption. 2019.

[35] Varnish C, Phillips AR, Laxman ST, Maxwell N, Halligan SL, Button KS. The relationship between placement instability and mental health among care-experienced children and young people: UK systematic review and meta-analysis. Br J Psychiatry. 2025;25:1–10. 10.1192/bjp.2025.10375. 10.1192/bjp.2025.10375 40993983

[36] Wade C. Trajectories for children and young people who experience out of home care: examining the influences of pre-care characteristics on later wellbeing and placement stability. Child Abuse Negl. 2024;149:106398. 10.1016/j.chiabu.2023.106398. 37612203 10.1016/j.chiabu.2023.106398

[37] Adoption UK. The Adoption Barometer A stocktake of adoption in Wales. 2024. Report No. Available from: https://www.adoptionuk.org/Handlers/Download.ashx?IDMF=b7422cbe-6407-4c43-a787-47076ef666ca.

[38] Palacios J, Rolock N, Selwyn J, Barbosa-Ducharne M. Adoption breakdown: concept, research, and implications. Res Soc Work Pract. 2019;29(2):130–42. 10.1177/1049731518783852.

[39] Hammond SP, Young J, Duddy C. Life story work for children and young people with care experience: a scoping review. Dev Child Welf. 2020;2(4):293–315. 10.1177/2516103220985872.

[40] Brown A, Shelton K. Coherent lives: making sense of adoptees’ experiences in education through narrative identity. Br Educ Res J. 2024;50(2):495–512. 10.1002/berj.3918.

[41] Woolgar M, Pinto C, González RA. Adoptive parents’ satisfaction with child and adolescent mental services and their mental health concerns over time: a question of fit? Dev Child Welf. 2024;6(1):50–64. 10.1177/25161032231221727.

[42] Sebba J, Berridge D, Luke N, Fletcher J, Bell K, Strand S, et al. The educational progress of looked after children in England: linking care and educational data. Bristol: Rees Centre/University of Bristol; 2015.

[43] MacDonald M, McSherry D. Open adoption: adoptive parents’ experiences of birth family contact and talking to their child about adoption. Adoption Fost. 2011;35(3):4–16. 10.1177/030857591103500302.

[44] Neil E, Beek M, Ward E. Contact After Adoption: A follow up in late adolescence. 2013.

[45] Hiller RM, Lehmann S, Lewis SJ, Minnis H, Shelton KH, Tarren-Sweeney M, et al. Accommodating complexity: the need for evidence-informed mental health assessments for children in out-of-home care. J Am Acad Child Adolesc Psychiatry. 2023;62(1):12–8. 10.1016/j.jaac.2022.06.021. 35987299 10.1016/j.jaac.2022.06.021

[46] Burch K, Backinsell A, Daru J, Halford E. Evaluation of the Adoption Support Fund: baseline survey of families. 2021.

[47] Burch K, Backinsell A. Evaluation of the Adoption Support Fund: first follow up survey of parents and carers. 2022.

[48] Burch K, Backinsell A. Evaluation of the Adoption Support Fund: second follow up survey of parents and carers. 2022.

[49] Sprecher E, Shelton K, Holmes L, Carter B, Robinson C, Javed M, et al. Sufficiency of current practice: How well does the Strengths and Difficulties Questionnaire detect clinically elevated posttraumatic stress, anxiety, and depression symptoms in children in care? J Psychol Psychiatry Adv. 2025. Available from: https://acamh.onlinelibrary.wiley.com/doi/full/10.1002/jcv2.70058. Cited 2026 Jan 17. 10.1002/jcv2.70058 PMC13260667 42291683

[50] MacDonald S, Hewitt G, Jones S, Rees A, Brown R, Anthony R, et al. Mental wellbeing needs and support for care-experienced children and young people in secondary school and during the transition to further education college. Child Soc. 2025;39(4):807–24. 10.1111/chso.12951.

