# Supplementary material for: A comparative study of mental health and wellbeing in adopted children, children in foster and kinship care, and the general population
Source: BMC Pediatr. 2026 Aug 21;26:769. doi: 10.1186/s12887-026-07104-x (PMC13495433; doi:10.1186/s12887-026-07104-x)
Supplement: Supplementary file 1 [file 12887_2026_7104_MOESM1_ESM.docx]

**Additional file 1**

**Frequency table of previous time living away from current home, for children in general population group**

| **Past living arrangement** | **Frequency** | **%** |
| --- | --- | --- |
| Never away from current home | 78750 | 77.8% |
| Other birth parent | 2565 | 2.5% |
| Kinship | 11802 | 11.7% |
| Time in care | 568 | 0.6% |
| No response / refusal to answer | 8938 | 8.8% |
| Total | 101187 |  |

*Responses not mutually exclusive for other birth parent, kinship and time in care

The table concerns the general population group only, and if they have spent time away from their current home. The table reduces the past living arrangement question to five subgroups, the first being children who have never spent more than a month away from their current home. The second group includes children who lived with another parent, and the third other kin (grandparent, aunt or uncle, or older sibling) for a month long period or longer. The fourth include children that have spent any time in care (residential, fostering or adoptive family). As with the analysis in the paper, children who were in independent living or living with someone else are not included in the table.
