# Supplementary material for: A comparative study of mental health and wellbeing in adopted children, children in foster and kinship care, and the general population
Source: BMC Pediatr. 2026 Aug 21;26:769. doi: 10.1186/s12887-026-07104-x (PMC13495433; doi:10.1186/s12887-026-07104-x)
Supplement: Supplementary file 2 [file 12887_2026_7104_MOESM2_ESM.docx]

**Additional file 2**

**Details of the development of living arrangements variable**

The original living arrangements variable included 13 options, with children able to select as many options as were applicable to them. Following the removal of the residential care subgroup due to the group being too small to include, as well as the independent living, living with someone else and the refusal to answer options, 9 remained.

The large number of response options resulted in inconsistencies that needed resolution, and this was achieved through reasoning the true living arrangements where possible. For example, children who were living with wider family members (i.e. aunt or uncle, grandparent or older sibling) but who also selected a care setting were treated as in familial foster care and included in the kinship group. Similarly, children who reported living with an adoptive family but also with a mother or father were reasoned to have developed a positive relationship with their adoptive parents, with this the same for children who were both fostered and with either or both parents.

When reasoning their true circumstances was not possible cases were excluded. This included children who selected that they were currently living in multiple care settings (i.e. at least two of residential, foster care or adoption) as their current home was unclear. Children who selected living with both stepparents and adopted families were also excluded as there was uncertainty over whether they had been adopted from care, or if they were living in a stepfamily and had not been previously looked after.
