# Supplementary material for: A comparative study of mental health and wellbeing in adopted children, children in foster and kinship care, and the general population
Source: BMC Pediatr. 2026 Aug 21;26:769. doi: 10.1186/s12887-026-07104-x (PMC13495433; doi:10.1186/s12887-026-07104-x)
Supplement: Supplementary file 3 [file 12887_2026_7104_MOESM3_ESM.docx]

**Additional file 3**

**Table of correlations.**

Multiple imputation was used to resolve missing data that was due to non-response. Estimations of missing values for each indicator were informed by the other variables that the indicator correlated with. Below is the table of correlations which indicates multi-collinearity between the sex and gender variables. This was expected due to the close relationship between these variables.

1. gender; 2. sexbirth; 3. grade; 4. fasfamcar; 5. fasbedroom; 6. fascomputers; 7. fasdishwash; 8. fasbathroom; 9. fasholidays; 10. WEMWBS_1; 11. WEMWBS_2; 12. WEMWBS_3; 13. WEMWBS_4; 14. WEMWBS_5; 15. WEMWBS_6; 16. WEMWBS_7; 17. SDQ1; 18. SDQ2; 19. SDQ3; 20. SDQ4; 21. SDQ5; 22. SDQ6; 23. SDQ7; 24. SDQ8; 25. SDQ9; 26. SDQ10; 27. SDQ11; 28. SDQ12; 29. SDQ13; 30. SDQ14; 31. SDQ15; 32. SDQ16; 33. SDQ17; 34. SDQ18; 35. SDQ19; 36. SDQ20; 37. SDQ21; 38. SDQ22; 39. SDQ23; 40. SDQ24; 41. SDQ25; 42; MFQ1; 43. MFQ2; 44. MFQ3; 45. MFQ4 ; 46. MFQ5; 47. MFQ6; 48. MFQ7; 49. MFQ8; 50. MFQ9; 51. MFQ10; 52. MFQ11; 53. MFQ12; 54. MFQ13; 55. NVwholivewith; 56. Ethnic.
