# Supplementary material for: A comparative study of mental health and wellbeing in adopted children, children in foster and kinship care, and the general population
Source: BMC Pediatr. 2026 Aug 21;26:769. doi: 10.1186/s12887-026-07104-x (PMC13495433; doi:10.1186/s12887-026-07104-x)
Supplement: Supplementary file 4 [file 12887_2026_7104_MOESM4_ESM.docx]

**Additional file 4**

**Full multi-level regression models, including standardised values**

|  | **Estimate** | **SE** | ***t*** | ***p*** | **LCI** | **UCI** | **Std_Estimate** |
| --- | --- | --- | --- | --- | --- | --- | --- |
| **SDQ total difficulties** | | |  |  |  |  |  |
| (Intercept) | 13.28 | 0.17 | 77.52 | 0.00 | 12.94 | 13.61 | NA |
| Kinship | 1.91 | 0.19 | 10.30 | 0.00 | 1.55 | 2.27 | 0.13 |
| Fostering | 1.00 | 0.29 | 3.44 | 0.00 | 0.43 | 1.57 | 0.07 |
| Adopted | 3.53 | 0.38 | 9.17 | 0.00 | 2.78 | 4.29 | 0.24 |
| Boy | -2.66 | 0.04 | -60.73 | 0.00 | -2.75 | -2.58 | -0.18 |
| Neither (gender) | 3.70 | 0.19 | 18.97 | 0.00 | 3.32 | 4.08 | 0.25 |
| School year | 0.57 | 0.02 | 36.26 | 0.00 | 0.54 | 0.60 | 0.04 |
| Asian/Asian British | -2.14 | 0.11 | -18.93 | 0.00 | -2.37 | -1.92 | -0.15 |
| Mixed or multiple | 0.27 | 0.13 | 2.12 | 0.03 | 0.02 | 0.52 | 0.02 |
| Other (ethnicity) | -0.98 | 0.17 | -5.88 | 0.00 | -1.31 | -0.65 | -0.07 |
| Black/Black British | -2.62 | 0.17 | -15.67 | 0.00 | -2.95 | -2.29 | -0.18 |
| Affluence | -0.32 | 0.01 | -35.47 | 0.00 | -0.34 | -0.31 | -0.11 |
|  |  |  |  |  |  |  |  |
|  |  |  |  |  |  |  |  |
| **SDQ internalising difficulties** | | |  |  |  |  |  |
| (Intercept) | 6.91 | 0.09 | 73.91 | 0.00 | 6.72 | 7.09 | NA |
| Kinship | 0.76 | 0.10 | 7.44 | 0.00 | 0.56 | 0.96 | 0.09 |
| Fostering | 0.48 | 0.16 | 3.01 | 0.00 | 0.17 | 0.80 | 0.06 |
| Adopted | 1.53 | 0.21 | 7.22 | 0.00 | 1.12 | 1.95 | 0.19 |
| Boy | -2.05 | 0.02 | -84.75 | 0.00 | -2.09 | -2.00 | -0.25 |
| Neither (gender) | 2.04 | 0.11 | 18.99 | 0.00 | 1.83 | 2.25 | 0.25 |
| School year | 0.28 | 0.01 | 32.12 | 0.00 | 0.26 | 0.29 | 0.03 |
| Asian/Asian British | -0.73 | 0.06 | -11.87 | 0.00 | -0.85 | -0.61 | -0.09 |
| Mixed or multiple | -0.03 | 0.07 | -0.47 | 0.64 | -0.17 | 0.11 | 0.00 |
| Other (ethnicity) | -0.42 | 0.09 | -4.64 | 0.00 | -0.60 | -0.24 | -0.05 |
| Black/Black British | -1.18 | 0.09 | -12.93 | 0.00 | -1.36 | -1.01 | -0.15 |
| Affluence | -0.21 | 0.01 | -41.76 | 0.00 | -0.22 | -0.20 | -0.13 |
|  |  |  |  |  |  |  |  |
|  |  |  |  |  |  |  |  |
| **SDQ externalising difficulties** | | |  |  |  |  |  |
| (Intercept) | 6.38 | 0.10 | 62.49 | 0.00 | 6.18 | 6.58 | NA |
| Kinship | 1.15 | 0.11 | 10.36 | 0.00 | 0.93 | 1.37 | 0.13 |
| Fostering | 0.52 | 0.17 | 2.96 | 0.00 | 0.17 | 0.86 | 0.06 |
| Adopted | 2.00 | 0.23 | 8.66 | 0.00 | 1.54 | 2.45 | 0.23 |
| Boy | -0.62 | 0.03 | -23.44 | 0.00 | -0.67 | -0.56 | -0.07 |
| Neither (gender) | 1.66 | 0.12 | 14.35 | 0.00 | 1.44 | 1.89 | 0.19 |
| School year | 0.29 | 0.01 | 30.96 | 0.00 | 0.27 | 0.31 | 0.03 |
| Asian/Asian British | -1.43 | 0.07 | -21.03 | 0.00 | -1.56 | -1.29 | -0.17 |
| Mixed or multiple | 0.30 | 0.08 | 3.98 | 0.00 | 0.15 | 0.45 | 0.04 |
| Other (ethnicity) | -0.57 | 0.10 | -5.73 | 0.00 | -0.76 | -0.37 | -0.07 |
| Black/Black British | -1.44 | 0.10 | -14.42 | 0.00 | -1.64 | -1.25 | -0.17 |
| Affluence | -0.11 | 0.01 | -21.02 | 0.00 | -0.13 | -0.10 | -0.07 |

|  | **Estimate** | **SE** | ***t*** | ***p*** | **LCI** | **UCI** | **Std_Estimate** |
| --- | --- | --- | --- | --- | --- | --- | --- |
| **SDQ prosocial** | |  |  |  |  |  |  |
| (Intercept) | 8.36 | 0.06 | 143.32 | 0.00 | 8.25 | 8.48 | NA |
| Kinship | -0.56 | 0.06 | -8.88 | 0.00 | -0.68 | -0.43 | -0.11 |
| Fostering | -0.54 | 0.10 | -5.50 | 0.00 | -0.73 | -0.35 | -0.11 |
| Adopted | -0.65 | 0.13 | -4.98 | 0.00 | -0.90 | -0.39 | -0.13 |
| Boy | -0.96 | 0.01 | -65.34 | 0.00 | -0.99 | -0.94 | -0.20 |
| Neither (gender) | -1.31 | 0.07 | -20.02 | 0.00 | -1.44 | -1.18 | -0.27 |
| School year | -0.19 | 0.01 | -35.31 | 0.00 | -0.20 | -0.18 | -0.04 |
| Asian/Asian British | -0.15 | 0.04 | -4.06 | 0.00 | -0.23 | -0.08 | -0.03 |
| Mixed or multiple | -0.22 | 0.04 | -5.08 | 0.00 | -0.30 | -0.13 | -0.04 |
| Other (ethnicity) | -0.15 | 0.05 | -2.75 | 0.01 | -0.26 | -0.04 | -0.03 |
| Black/Black British | -0.13 | 0.06 | -2.18 | 0.03 | -0.25 | -0.01 | -0.03 |
| Affluence | 0.07 | 0.00 | 23.08 | 0.00 | 0.07 | 0.08 | 0.07 |
|  |  |  |  |  |  |  |  |
|  |  |  |  |  |  |  |  |
| **Wellbeing** | |  |  |  |  |  |  |
| (Intercept) | 18.43 | 0.14 | 129.18 | 0.00 | 18.15 | 18.71 | NA |
| Kinship | -1.83 | 0.16 | -11.77 | 0.00 | -2.14 | -1.53 | -0.15 |
| Fostering | -1.76 | 0.24 | -7.23 | 0.00 | -2.24 | -1.29 | -0.14 |
| Adopted | -3.12 | 0.32 | -9.63 | 0.00 | -3.75 | -2.48 | -0.25 |
| Boy | 2.31 | 0.04 | 62.29 | 0.00 | 2.23 | 2.38 | 0.19 |
| Neither (gender) | -3.02 | 0.17 | -18.28 | 0.00 | -3.34 | -2.69 | -0.24 |
| School year | -0.10 | 0.01 | -7.65 | 0.00 | -0.13 | -0.07 | -0.01 |
| Asian/Asian British | 0.45 | 0.10 | 4.46 | 0.00 | 0.25 | 0.64 | 0.04 |
| Mixed or multiple | -0.10 | 0.11 | -0.97 | 0.33 | -0.31 | 0.11 | -0.01 |
| Other (ethnicity) | -0.06 | 0.14 | -0.42 | 0.68 | -0.33 | 0.21 | -0.01 |
| Black/Black British | 1.60 | 0.15 | 10.33 | 0.00 | 1.29 | 1.90 | 0.13 |
| Affluence | 0.46 | 0.01 | 60.45 | 0.00 | 0.45 | 0.48 | 0.19 |
|  |  |  |  |  |  |  |  |
|  |  |  |  |  |  |  |  |
| **MFQ** |  |  |  |  |  |  |  |
| (Intercept) | 4.45 | 0.29 | 15.15 | 0.00 | 3.87 | 5.02 | NA |
| Kinship | 0.91 | 0.12 | 7.52 | 0.00 | 0.67 | 1.14 | 0.07 |
| Fostering | 0.51 | 0.19 | 2.68 | 0.01 | 0.14 | 0.88 | 0.04 |
| Adopted | 1.48 | 0.25 | 5.92 | 0.00 | 0.99 | 1.97 | 0.12 |
| Boy | -1.93 | 0.03 | -67.50 | 0.00 | -1.98 | -1.87 | -0.16 |
| Neither (gender) | 1.90 | 0.13 | 15.08 | 0.00 | 1.66 | 2.15 | 0.16 |
| School year | 0.17 | 0.01 | 17.01 | 0.00 | 0.15 | 0.19 | 0.01 |
| Asian/Asian British | -0.28 | 0.07 | -3.77 | 0.00 | -0.42 | -0.13 | -0.02 |
| Mixed or multiple | 0.23 | 0.08 | 2.91 | 0.00 | 0.08 | 0.39 | 0.02 |
| Other (ethnicity) | -0.05 | 0.11 | -0.45 | 0.65 | -0.26 | 0.16 | 0.00 |
| Black/Black British | -0.67 | 0.11 | -6.17 | 0.00 | -0.88 | -0.46 | -0.06 |
| Affluence | -0.15 | 0.01 | -25.15 | 0.00 | -0.16 | -0.14 | -0.06 |
