# Supplementary material for: A comparative study of mental health and wellbeing in adopted children, children in foster and kinship care, and the general population
Source: BMC Pediatr. 2026 Aug 21;26:769. doi: 10.1186/s12887-026-07104-x (PMC13495433; doi:10.1186/s12887-026-07104-x)
Supplement: Supplementary file 5 [file 12887_2026_7104_MOESM5_ESM.docx]

**Additional file 5**

**List-wise analysis for comparison to models with imputed data.**

The table presents the model results for complete cases only, for the purpose of comparison. Any cases with missing demographic data were excluded from the analysis completely. Cases with a missing outcomes indicator were not included in that part of the analysis (eg WEMWEBS_1 and the wellbeing model)

|  | **Estimate** | **SE** | ***t*** | ***p*** | **LCI** | **UCI** |
| --- | --- | --- | --- | --- | --- | --- |
| **SDQ total difficulties** | |  |  |  |  |  |
| (Intercept) | 14.71 | 0.19 | 78.43 | 0.00 | 14.35 | 15.08 |
| Kinship | 2.48 | 0.21 | 12.02 | 0.00 | 2.08 | 2.89 |
| Fostering | 1.63 | 0.33 | 4.90 | 0.00 | 0.98 | 2.28 |
| Adopted | 4.15 | 0.44 | 9.35 | 0.00 | 3.28 | 5.02 |
| Boy | -2.70 | 0.05 | -58.61 | 0.00 | -2.79 | -2.61 |
| Neither (gender) | 4.64 | 0.23 | 20.61 | 0.00 | 4.20 | 5.09 |
| School year | 0.55 | 0.02 | 33.09 | 0.00 | 0.52 | 0.58 |
| Asian/Asian British | -2.40 | 0.18 | -13.24 | 0.00 | -2.75 | -2.04 |
| Mixed or multiple | -2.16 | 0.12 | -18.41 | 0.00 | -2.39 | -1.93 |
| Other (ethnicity) | 0.32 | 0.13 | 2.50 | 0.01 | 0.07 | 0.57 |
| Black/Black British | -0.90 | 0.17 | -5.19 | 0.00 | -1.24 | -0.56 |
| Affluence | -0.41 | 0.01 | -40.38 | 0.00 | -0.43 | -0.39 |
|  |  |  |  |  |  |  |
| **SDQ internalising difficulties** | | |  |  |  |  |
| (Intercept) | 7.54 | 0.10 | 73.49 | 0.00 | 7.34 | 7.74 |
| Kinship | 0.97 | 0.11 | 8.52 | 0.00 | 0.75 | 1.19 |
| Fostering | 0.72 | 0.18 | 3.96 | 0.00 | 0.36 | 1.08 |
| Adopted | 1.82 | 0.24 | 7.52 | 0.00 | 1.34 | 2.29 |
| Boy | -2.12 | 0.03 | -83.18 | 0.00 | -2.17 | -2.07 |
| Neither (gender) | 2.55 | 0.12 | 20.74 | 0.00 | 2.31 | 2.79 |
| School year | 0.27 | 0.01 | 29.85 | 0.00 | 0.26 | 0.29 |
| Asian/Asian British | -1.10 | 0.10 | -10.95 | 0.00 | -1.29 | -0.90 |
| Mixed or multiple | -0.71 | 0.06 | -11.01 | 0.00 | -0.84 | -0.58 |
| Other (ethnicity) | -0.04 | 0.07 | -0.59 | 0.56 | -0.18 | 0.10 |
| Black/Black British | -0.38 | 0.09 | -4.03 | 0.00 | -0.57 | -0.20 |
| Affluence | -0.25 | 0.01 | -44.56 | 0.00 | -0.26 | -0.24 |
|  |  |  |  |  |  |  |
| **SDQ externalising difficulties** | | |  |  |  |  |
| (Intercept) | 7.28 | 0.11 | 64.89 | 0.00 | 7.06 | 7.50 |
| Kinship | 1.50 | 0.12 | 12.04 | 0.00 | 1.26 | 1.74 |
| Fostering | 0.92 | 0.20 | 4.65 | 0.00 | 0.53 | 1.31 |
| Adopted | 2.30 | 0.26 | 8.69 | 0.00 | 1.78 | 2.82 |
| Boy | -0.56 | 0.03 | -20.30 | 0.00 | -0.62 | -0.51 |
| Neither (gender) | 2.11 | 0.13 | 15.72 | 0.00 | 1.85 | 2.38 |
| School year | 0.26 | 0.01 | 26.37 | 0.00 | 0.24 | 0.28 |
| Asian/Asian British | -1.33 | 0.11 | -12.09 | 0.00 | -1.54 | -1.11 |
| Mixed or multiple | -1.44 | 0.07 | -20.52 | 0.00 | -1.58 | -1.31 |
| Other (ethnicity) | 0.37 | 0.08 | 4.73 | 0.00 | 0.22 | 0.52 |
| Black/Black British | -0.48 | 0.10 | -4.63 | 0.00 | -0.69 | -0.28 |
| Affluence | -0.16 | 0.01 | -25.89 | 0.00 | -0.17 | -0.15 |

|  | **Estimate** | **SE** | ***t*** | ***p*** | **LCI** | **UCI** |
| --- | --- | --- | --- | --- | --- | --- |
| **SDQ prosocial** |  |  |  |  |  |  |
| (Intercept) | 0.57 | 0.05 | 10.56 | 0.00 | 0.47 | 0.68 |
| Kinship | 0.41 | 0.06 | 6.79 | 0.00 | 0.29 | 0.52 |
| Fostering | 0.28 | 0.10 | 2.96 | 0.00 | 0.10 | 0.47 |
| Adopted | 0.48 | 0.12 | 3.88 | 0.00 | 0.24 | 0.73 |
| Boy | 0.90 | 0.01 | 66.45 | 0.00 | 0.87 | 0.92 |
| Neither (gender) | 1.08 | 0.06 | 16.56 | 0.00 | 0.95 | 1.20 |
| School year | 0.24 | 0.00 | 49.99 | 0.00 | 0.23 | 0.25 |
| Asian/Asian British | 0.01 | 0.05 | 0.10 | 0.92 | -0.10 | 0.11 |
| Mixed or multiple | 0.12 | 0.03 | 3.50 | 0.00 | 0.05 | 0.19 |
| Other (ethnicity) | 0.22 | 0.04 | 5.75 | 0.00 | 0.14 | 0.29 |
| Black/Black British | 0.05 | 0.05 | 1.01 | 0.31 | -0.05 | 0.15 |
| Affluence | -0.04 | 0.00 | -14.59 | 0.00 | -0.05 | -0.04 |
|  |  |  |  |  |  |  |
| **Wellbeing** |  |  |  |  |  |  |
| (Intercept) | 21.76 | 0.14 | 157.94 | 0.00 | 21.48 | 22.02 |
| Kinship | -1.63 | 0.15 | -10.71 | 0.00 | -1.93 | -1.33 |
| Fostering | -1.08 | 0.24 | -4.41 | 0.00 | -1.56 | -0.60 |
| Adopted | -2.74 | 0.32 | -8.52 | 0.00 | -3.36 | -2.11 |
| Boy | 2.41 | 0.03 | 69.94 | 0.00 | 2.34 | 2.48 |
| Neither (gender) | -2.91 | 0.17 | -17.59 | 0.00 | -3.23 | -2.58 |
| School year | -0.28 | 0.01 | -22.55 | 0.00 | -0.30 | -0.25 |
| Asian/Asian British | 1.58 | 0.13 | 11.83 | 0.00 | 1.32 | 1.85 |
| Mixed or multiple | 0.59 | 0.09 | 6.78 | 0.00 | 0.42 | 0.76 |
| Other (ethnicity) | -0.18 | 0.10 | -1.88 | 0.06 | -0.37 | 0.01 |
| Black/Black British | 0.22 | 0.13 | 1.71 | 0.09 | -0.03 | 0.47 |
| Affluence | 0.35 | 0.01 | 46.39 | 0.00 | 0.33 | 0.36 |
|  |  |  |  |  |  |  |
| **MFQ** |  |  |  |  |  |  |
| (Intercept) | 9.36 | 0.24 | 38.37 | 0.00 | 8.88 | 9.83 |
| Kinship | 1.85 | 0.26 | 7.16 | 0.00 | 1.34 | 2.36 |
| Fostering | 0.96 | 0.42 | 2.27 | 0.02 | 0.13 | 1.79 |
| Adopted | 4.24 | 0.58 | 7.29 | 0.00 | 3.10 | 5.38 |
| Boy | -4.07 | 0.06 | -67.97 | 0.00 | -4.18 | -3.95 |
| Neither (gender) | 4.71 | 0.29 | 16.15 | 0.00 | 4.14 | 5.29 |
| School year | 0.38 | 0.02 | 17.86 | 0.00 | 0.34 | 0.43 |
| Asian/Asian British | -1.28 | 0.23 | -5.59 | 0.00 | -1.73 | -0.83 |
| Mixed or multiple | -0.58 | 0.15 | -3.92 | 0.00 | -0.87 | -0.29 |
| Other (ethnicity) | 0.54 | 0.17 | 3.22 | 0.00 | 0.21 | 0.87 |
| Black/Black British | -0.19 | 0.23 | -0.84 | 0.40 | -0.65 | 0.26 |
| Affluence | -0.35 | 0.01 | -26.60 | 0.00 | -0.37 | -0.32 |
