# Supplementary material for: A comparative study of mental health and wellbeing in adopted children, children in foster and kinship care, and the general population
Source: BMC Pediatr. 2026 Aug 21;26:769. doi: 10.1186/s12887-026-07104-x (PMC13495433; doi:10.1186/s12887-026-07104-x)
Supplement: Supplementary file 6 [file 12887_2026_7104_MOESM6_ESM.docx]

**Additional file 6**

**Multi-level model results with the residential subgroup included.**

S6 presents the models results for the imputed data with the residential subgroup included. This group was excluded from the analysis reported in the paper due to the size of the subsample.

|  | **Estimate** | **SE** | ***t*** | ***p*** | **LCI** | **UCI** | **Std_Estimate** |
| --- | --- | --- | --- | --- | --- | --- | --- |
| **SDQ total difficulties** | |  |  |  |  |  |  |
| (Intercept) | 13.27 | 0.17 | 77.47 | 0.00 | 12.94 | 13.61 | NA |
| Kinship | 1.91 | 0.19 | 10.28 | 0.00 | 1.54 | 2.27 | 0.13 |
| Fostering | 1.02 | 0.29 | 3.51 | 0.00 | 0.45 | 1.59 | 0.07 |
| Adopted | 3.54 | 0.39 | 9.17 | 0.00 | 2.78 | 4.29 | 0.24 |
| Residential | 1.29 | 0.66 | 1.95 | 0.05 | -0.01 | 2.59 | 0.09 |
| Boy | -2.67 | 0.04 | -60.46 | 0.00 | -2.75 | -2.58 | -0.18 |
| Neither (gender) | 3.63 | 0.19 | 18.91 | 0.00 | 3.26 | 4.01 | 0.25 |
| School year | 0.57 | 0.02 | 36.27 | 0.00 | 0.54 | 0.60 | 0.04 |
| Asian/Asian British | -2.12 | 0.11 | -19.24 | 0.00 | -2.34 | -1.91 | -0.15 |
| Mixed or multiple | 0.28 | 0.12 | 2.32 | 0.02 | 0.04 | 0.52 | 0.02 |
| Other (ethnicity) | -1.01 | 0.16 | -6.26 | 0.00 | -1.32 | -0.69 | -0.07 |
| Black/Black British | -2.59 | 0.17 | -15.67 | 0.00 | -2.91 | -2.26 | -0.18 |
| Affluence | -0.32 | 0.01 | -35.48 | 0.00 | -0.34 | -0.31 | -0.11 |
| ICC: | 0.009 |  |  |  |  |  |  |
|  |  |  |  |  |  |  |  |
| **SDQ Internalising** | |  |  |  |  |  |  |
| (Intercept) | 6.91 | 0.09 | 73.86 | 0.00 | 6.72 | 7.09 | NA |
| Kinship | 0.76 | 0.10 | 7.41 | 0.00 | 0.56 | 0.96 | 0.09 |
| Fostering | 0.49 | 0.16 | 3.08 | 0.00 | 0.18 | 0.81 | 0.06 |
| Adopted | 1.54 | 0.21 | 7.22 | 0.00 | 1.12 | 1.95 | 0.19 |
| Residential | 0.41 | 0.37 | 1.13 | 0.26 | -0.30 | 1.13 | 0.05 |
| Boy | -2.05 | 0.02 | -84.14 | 0.00 | -2.09 | -2.00 | -0.25 |
| Neither (gender) | 2.00 | 0.11 | 18.55 | 0.00 | 1.79 | 2.22 | 0.25 |
| School year | 0.28 | 0.01 | 32.12 | 0.00 | 0.26 | 0.29 | 0.03 |
| Asian/Asian British | -0.73 | 0.06 | -11.90 | 0.00 | -0.85 | -0.61 | -0.09 |
| Mixed or multiple | -0.03 | 0.07 | -0.37 | 0.71 | -0.16 | 0.11 | 0.00 |
| Other (ethnicity) | -0.44 | 0.09 | -4.83 | 0.00 | -0.61 | -0.26 | -0.05 |
| Black/Black British | -1.16 | 0.09 | -12.62 | 0.00 | -1.35 | -0.98 | -0.14 |
| Affluence | -0.21 | 0.01 | -41.76 | 0.00 | -0.22 | -0.20 | -0.13 |
| ICC: | 0.007 |  |  |  |  |  |  |

|  | **Estimate** | **SE** | ***t*** | ***p*** | **LCI** | **UCI** | **Std_Estimate** |
| --- | --- | --- | --- | --- | --- | --- | --- |
| **SDQ Externalising** | |  |  |  |  |  |  |
| (Intercept) | 6.37 | 0.10 | 62.45 | 0.00 | 6.17 | 6.57 | NA |
| Kinship | 1.15 | 0.11 | 10.35 | 0.00 | 0.93 | 1.37 | 0.13 |
| Fostering | 0.52 | 0.17 | 3.01 | 0.00 | 0.18 | 0.87 | 0.06 |
| Adopted | 2.00 | 0.23 | 8.66 | 0.00 | 1.55 | 2.45 | 0.23 |
| Residential | 0.88 | 0.40 | 2.21 | 0.03 | 0.10 | 1.65 | 0.10 |
| Boy | -0.62 | 0.03 | -23.48 | 0.00 | -0.67 | -0.57 | -0.07 |
| Neither (gender) | 1.63 | 0.11 | 14.32 | 0.00 | 1.41 | 1.85 | 0.19 |
| School year | 0.29 | 0.01 | 30.99 | 0.00 | 0.27 | 0.31 | 0.03 |
| Asian/Asian British | -1.41 | 0.07 | -21.28 | 0.00 | -1.54 | -1.28 | -0.16 |
| Mixed or multiple | 0.31 | 0.07 | 4.16 | 0.00 | 0.16 | 0.45 | 0.04 |
| Other (ethnicity) | -0.58 | 0.10 | -6.05 | 0.00 | -0.76 | -0.39 | -0.07 |
| Black/Black British | -1.43 | 0.10 | -14.55 | 0.00 | -1.62 | -1.24 | -0.17 |
| Affluence | -0.11 | 0.01 | -21.03 | 0.00 | -0.13 | -0.10 | -0.07 |
| ICC: | 0.008 |  |  |  |  |  |  |
|  |  |  |  |  |  |  |  |
| **SDQ Prosocial** |  |  |  |  |  |  |  |
| (Intercept) | 8.36 | 0.06 | 143.29 | 0.00 | 8.25 | 8.48 | NA |
| Kinship | -0.56 | 0.06 | -8.88 | 0.00 | -0.68 | -0.43 | -0.11 |
| Fostering | -0.54 | 0.10 | -5.49 | 0.00 | -0.73 | -0.35 | -0.11 |
| Adopted | -0.65 | 0.13 | -4.98 | 0.00 | -0.90 | -0.39 | -0.13 |
| Residential | -1.83 | 0.22 | -8.21 | 0.00 | -2.26 | -1.39 | -0.37 |
| Boy | -0.96 | 0.01 | -65.16 | 0.00 | -0.99 | -0.93 | -0.20 |
| Neither (gender) | -1.31 | 0.06 | -20.33 | 0.00 | -1.44 | -1.19 | -0.27 |
| School year | -0.19 | 0.01 | -35.31 | 0.00 | -0.20 | -0.18 | -0.04 |
| Asian/Asian British | -0.16 | 0.04 | -4.08 | 0.00 | -0.24 | -0.08 | -0.03 |
| Mixed or multiple | -0.21 | 0.04 | -5.16 | 0.00 | -0.29 | -0.13 | -0.04 |
| Other (ethnicity) | -0.16 | 0.06 | -2.73 | 0.01 | -0.27 | -0.04 | -0.03 |
| Black/Black British | -0.13 | 0.06 | -2.28 | 0.02 | -0.24 | -0.02 | -0.03 |
| Affluence | 0.07 | 0.00 | 23.12 | 0.00 | 0.07 | 0.08 | 0.07 |
| ICC: | 0.012 |  |  |  |  |  |  |

|  | **Estimate** | **SE** | ***t*** | ***p*** | **LCI** | **UCI** | **Std_Estimate** |
| --- | --- | --- | --- | --- | --- | --- | --- |
| **WEMWEBS** |  |  |  |  |  |  |  |
| (Intercept) | 18.42 | 0.14 | 129.39 | 0.00 | 18.14 | 18.70 | NA |
| Kinship | -1.83 | 0.16 | -11.73 | 0.00 | -2.13 | -1.52 | -0.15 |
| Fostering | -1.78 | 0.24 | -7.28 | 0.00 | -2.26 | -1.30 | -0.14 |
| Adopted | -3.11 | 0.32 | -9.63 | 0.00 | -3.75 | -2.48 | -0.25 |
| Residential | -3.40 | 0.56 | -6.12 | 0.00 | -4.49 | -2.31 | -0.28 |
| Boy | 2.31 | 0.04 | 62.68 | 0.00 | 2.24 | 2.38 | 0.19 |
| Neither (gender) | -3.02 | 0.16 | -18.89 | 0.00 | -3.33 | -2.71 | -0.24 |
| School year | -0.10 | 0.01 | -7.63 | 0.00 | -0.13 | -0.07 | -0.01 |
| Asian/Asian British | 0.43 | 0.10 | 4.22 | 0.00 | 0.23 | 0.63 | 0.03 |
| Mixed or multiple | -0.11 | 0.10 | -1.10 | 0.27 | -0.32 | 0.09 | -0.01 |
| Other (ethnicity) | -0.04 | 0.13 | -0.30 | 0.77 | -0.30 | 0.22 | 0.00 |
| Black/Black British | 1.57 | 0.14 | 10.85 | 0.00 | 1.28 | 1.85 | 0.13 |
| Affluence | 0.46 | 0.01 | 60.65 | 0.00 | 0.45 | 0.48 | 0.19 |
| ICC | 0.007 |  |  |  |  |  |  |
|  |  |  |  |  |  |  |  |
| **MFQ** |  |  |  |  |  |  |  |
| (Intercept) | 4.44 | 0.29 | 15.12 | 0.00 | 3.87 | 5.02 | NA |
| Kinship | 0.90 | 0.12 | 7.49 | 0.00 | 0.67 | 1.14 | 0.07 |
| Fostering | 0.51 | 0.19 | 2.72 | 0.01 | 0.14 | 0.89 | 0.04 |
| Adopted | 1.48 | 0.25 | 5.91 | 0.00 | 0.99 | 1.97 | 0.12 |
| Residential | 1.07 | 0.43 | 2.49 | 0.01 | 0.23 | 1.92 | 0.09 |
| Boy | -1.93 | 0.03 | -67.32 | 0.00 | -1.99 | -1.87 | -0.16 |
| Neither (gender) | 1.91 | 0.13 | 15.17 | 0.00 | 1.67 | 2.16 | 0.16 |
| School year | 0.17 | 0.01 | 17.00 | 0.00 | 0.15 | 0.19 | 0.01 |
| Asian/Asian British | -0.26 | 0.07 | -3.58 | 0.00 | -0.41 | -0.12 | -0.02 |
| Mixed or multiple | 0.24 | 0.08 | 2.86 | 0.00 | 0.08 | 0.41 | 0.02 |
| Other (ethnicity) | -0.04 | 0.11 | -0.41 | 0.68 | -0.25 | 0.17 | 0.00 |
| Black/Black British | -0.63 | 0.11 | -5.83 | 0.00 | -0.84 | -0.42 | -0.05 |
| Affluence | -0.15 | 0.01 | -25.04 | 0.00 | -0.16 | -0.14 | -0.06 |
| ICC: | 0.010 |  |  |  |  |  |  |
